# AJUBA and WTIP can compete with LIMD1 for junctional localization and LATS regulation

**DOI:** 10.17912/micropub.biology.000666

**Published:** 2022-11-09

**Authors:** Elmira Kirichenko, Kenneth D Irvine

**Affiliations:** 1 Waksman Institute and Department of Molecular Biology and Biochemistry, Rutgers University, 190 Frelinghusen Rd, Piscataway NJ 08854 USA

## Abstract

Each of the three mammalian Ajuba family proteins, AJUBA, LIMD1 and WTIP, exhibit tension-dependent localization to adherens junctions, and can associate with Lats kinases. However, only LIMD1 has been directly demonstrated to directly regulate Lats activity in vivo. To assess the relationship of LIMD1 to AJUBA and WTIP, and the potential contributions of AJUBA and WTIP to Lats regulation, we examined the consequences of over-expressing AJUBA and WTIP in MCF10A cells. Over-expression of either AJUBA or WTIP reduced junctional localization of LIMD1, implying that these proteins can compete for binding to adherens junctions. This over-expression also reduced junctional localization of LATS1, implying that AJUBA or WTIP are unable to efficiently recruit Lats kinases to adherens junctions. This over-expression was also associated with increased YAP1 phosphorylation and decreased YAP1 nuclear localization, consistent with increased Lats kinase activity. These observations indicate that AJUBA and WTIP compete with LIMD1 for association with adherens junctions but have activities distinct from LIMD1 in Hippo pathway regulation. They further suggest that the ability of Ajuba family proteins to associate with Lats kinases in solution is not sufficient to enable regulation in vivo, and that tumor suppressor activities of AJUBA and WTIP could stem in part from competition with LIMD1 for regulation of Lats kinases at cell junctions.

**Figure 1. Over-expression of AJUBA or WTIP reduces junctional localization of LIMD1 and LATS1, and decreases YAP1 activation:**
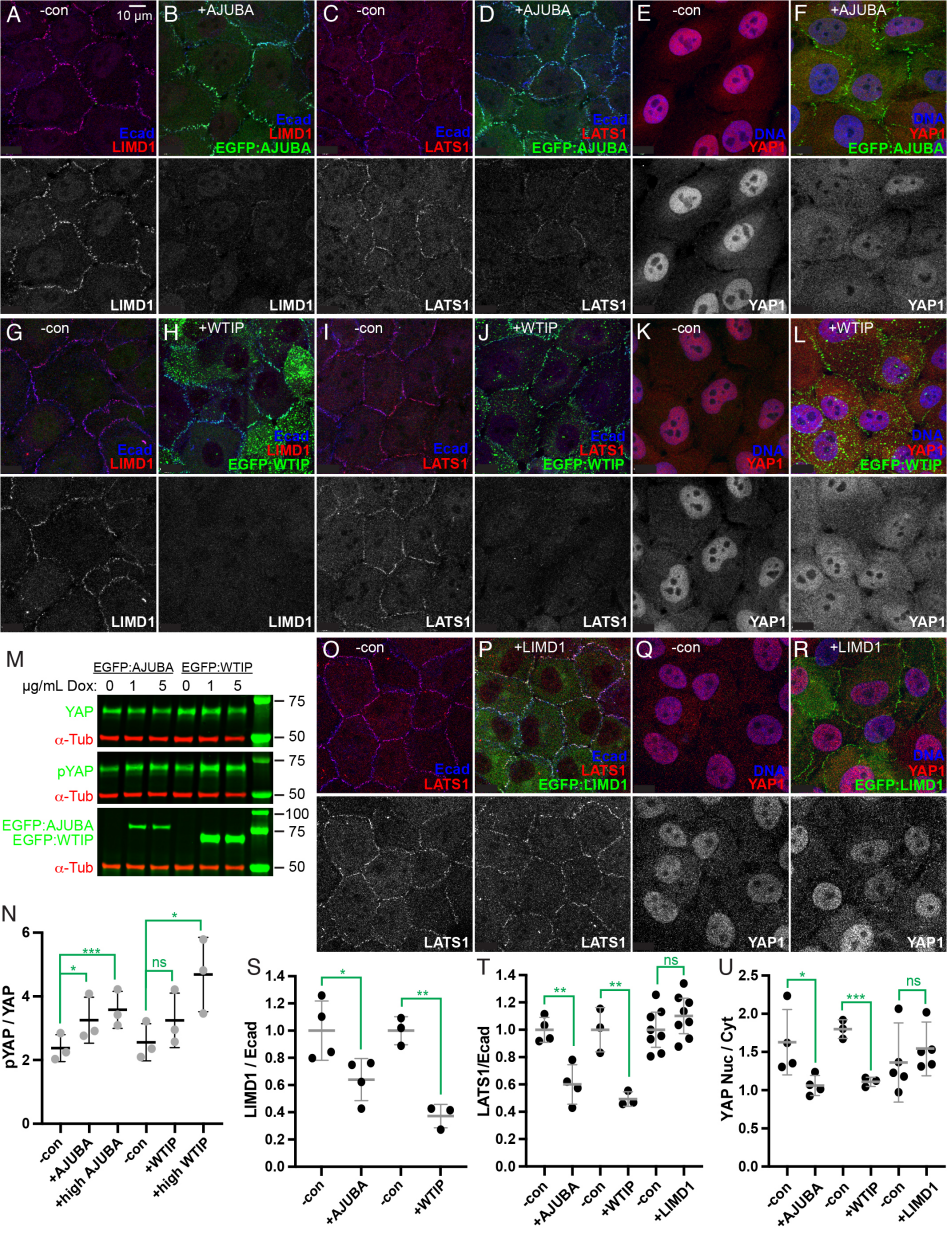
A-L) Representative examples of MCF10A cells, stained for E-cad (blue), DNA (using Hoechst, blue), LIMD1 (red/white), LATS1 (red/white), YAP1 (red/white), and expressing EGFP:AJUBA or EGFP:WTIP (green), as indicated. Panels below show a single channel in gray scale. (A-F) show cells with an inducible EGFP:AJUBA transgene, cultured in either 0 µg/mL Dox (-con, A,C,E) or 5 µg/mL Dox (+AJUBA, B,D,F). (G-L) show cells with inducible EGFP:WTIP transgene, cultured in either 0 µg/mL Dox (-con, G,I,K) or 5 µg/mL Dox (+WTIP, H,J,L). M) Representative western blots on lysates of MCF10A cells expressing EGFP:AJUBA or EGFP:WTIP and induced with 0, 1, or 5 µg/mL Dox, as indicated. Blots were stained for YAP1 (top), pYAP1 (middle) or GFP (bottom), together with α-Tubulin as a loading control. Bands at far right are molecular weight markers. N) Scatter plot showing quantitation of relative levels of pYAP/YAP in lysates of MCF10A cells. -con indicates cells treated with 0 µg/mL Dox, +AJUBA or +WTIP indicates cells treated with 1 µg/mL Dox, +high AJUBA or +high WTIP indicates cells treated with 5 µg/mL Dox. Error bars indicate standard deviation, and the significance of comparisons between values from induced and control cells are indicated in green. O-R) Representative examples of MCF10A cells, stained for E-cad (blue), DNA (using Hoechst, blue), LATS1 (red/white), YAP1 (red/white), and expressing EGFP:LIMD1 (green), as indicated and cultured in either 0 µg/mL Dox (-con, O,Q) or 5 µg/mL Dox (+LIMD1, P,R). Panels below show a single channel in gray scale. S) Scatter plot showing quantitation of relative levels (normalized to mean control values) of LIMD1 overlapping E-cad staining in MCF10A cells expressing EGFP:AJUBA or EGFP:WTIP, as in the examples above. T) Scatter plot showing quantitation of relative levels (normalized to mean control values) of LATS1 overlapping E-cad staining in MCF10A cells expressing EGFP:AJUBA, EGFP:WTIP, or EGFP:LIMD1, as in the examples above. U) Scatter plot showing quantitation of relative levels of nuclear YAP1/cytoplasmic YAP1 in MCF10A cells expressing EGFP:AJUBA, EGFP:WTIP, or EGFP:LIMD1, as in the examples above. For S-U), -con indicates cells treated with 0 µg/mL Dox, +AJUBA, +WTIP or +LIMD1 indicates cells treated with 5 µg/mL Dox. Error bars indicate standard deviation, and the significance of comparisons between values from induced and control cells are indicated in green.

## Description

**Introduction**

The Ajuba family of LIM domain proteins influence a variety of cellular processes (Schimizzi and Longmore, 2015; Schleicher and Schramek, 2021). One notable function is physical interaction with and inhibition of Lats family kinases (Abe et al., 2006; Codelia et al., 2014; Das Thakur et al., 2010; Ibar et al., 2018; Jagannathan et al., 2016; Rauskolb et al., 2011; Rauskolb et al., 2014; Reddy and Irvine, 2013; Sun and Irvine, 2013). Lats proteins (LATS1 and LATS2 in mammals, Warts in *Drosophila* ) are kinases that play key roles in Hippo signaling (Misra and Irvine, 2018; Zheng and Pan, 2019). Once activated by upstream cues, Lats kinases phosphorylate Yap family transcriptional co-activator proteins (YAP1 and TAZ in mammals, Yorkie in *Drosophila* ). Phosphorylation of Yap proteins promotes their cytoplasmic localization and degradation, whereas unphosphorylated Yap proteins can accumulate in the nucleus, where they promote transcription of downstream target genes.

Hippo signaling controls growth and cell fate in animals from *Drosophila* to humans (Misra and Irvine, 2018; Zanconato et al., 2016). Hippo signaling is regulated by a variety of upstream inputs, including cytoskeletal tension (Misra and Irvine, 2018; Sun and Irvine, 2016). Ajuba family proteins play a key role in one mechanism by which cytoskeletal tension modulates Hippo signaling. This was discovered in *Drosophila* wing imaginal discs, where the *Drosophila* Ajuba LIM protein (Jub) associates with an open form of α-catenin that is induced by tension at adherens junctions (AJ) (Alégot et al., 2019; Rauskolb *et al.* , 2014; Sarpal et al., 2019). Once bound to AJ, Jub then recruits and inhibits Warts. Recruitment of Warts to AJ by Jub in *Drosophila* sequesters Wts from upstream activators of Hippo signaling (Sun et al., 2015).

There are three mammalian Ajuba family proteins, AJUBA, WTIP, and LIMD1. Each of them can be recruited to AJ under tension (Ibar *et al.* , 2018). The recruitment and inhibition of Lats kinases at AJ cells when there is tension in the actin cytoskeleton is conserved in mammals (Dutta et al., 2018; Ibar *et al.* , 2018; Venkatramanan et al., 2021). All Ajuba family proteins are characterized by three conserved C-terminal LIM domains (Schimizzi and Longmore, 2015; Schleicher and Schramek, 2021). Structure-functions studies have revealed that the LIM domains play a key role in association of Ajuba family proteins with AJ, presumably due to association with an open form of α-catenin (Marie et al., 2003; Rauskolb et al., 2022; Rauskolb *et al.* , 2014; Razzell et al., 2018). Each of the mammalian Ajuba family proteins has also been reported to be able to associate with Lats kinases (Abe *et al.* , 2006; Das Thakur *et al.* , 2010; Jagannathan *et al.* , 2016; Reddy and Irvine, 2013; Sun and Irvine, 2013). Association of Ajuba family proteins with Lats kinases is also mediated by LIM domains (Jagannathan *et al.* , 2016; Rauskolb *et al.* , 2022). Since the LIM domains are conserved between AJUBA family proteins, this raises the question of whether they compete with each other for association with either AJ or Lats kinases, and if so, what are the functional consequences of this competition?

Although in some contexts it has been suggested that mammalian Ajuba family proteins act redundantly to regulate Lats kinases (Das Thakur *et al.* , 2010; Jagannathan *et al.* , 2016), knockdown experiments in confluent MCF10A cells cultured at low density revealed that LIMD1 is specifically required for tension-dependent recruitment of Lats kinases to AJ, and that this recruitment inhibits Lats activity (Ibar *et al.* , 2018). Conversely, AJUBA and WTIP were not required for localization or regulation of Lats kinases. Whether this reflects differences in expression levels amongst Ajuba family proteins, or differences in activity, was unclear. As one way to address this, we describe here investigations of the consequences of replacing LIMD1 at AJ with AJUBA or WTIP through over-expression of AJUBA or WTIP in MCF10A cells.

**Results**

To investigate the potential for competition between AJUBA or WTIP and LIMD1, we took advantage of cell lines expressing EGFP-tagged proteins that were created previously for analysis of the influence of cytoskeletal tension on the localization of Ajuba family proteins (Ibar *et al.* , 2018). For those prior experiments, cell lines transduced with Doxycycline (Dox)-inducible transgenes were created so that they could be expressed at or near endogenous levels. Western blotting with antibodies against AJUBA led to estimates that induction with 0.025 µg/mL Dox led to levels of EGFP:AJUBA comparable to endogenous AJUBA in an EGFP:AJUBA MCF10A cell line (Ibar *et al.* , 2018). Antibodies specifically recognizing WTIP were not available, so instead the lowest dose enabling readily detection of EGFP:WTIP in an EGFP:WTIP MCF10A cell line was used (0.05 µg/mL Dox). Induction of AJUBA or WTIP at these low levels did not have noticeable effects on Hippo signaling. For the present experiments, we wanted to use high, saturating levels of Dox that would induce maximal expression of these proteins, so we induced expression of EGFP:AJUBA or EGFP:WTIP using 5 µg/mL Dox, which based on earlier studies of AJUBA (Ibar *et al.* , 2018) is expected to generate expression levels well above endogenous levels.

***Over-expression of WTIP or AJUBA can displace LIMD1 from AJ***

MCF10A cells cultured at low cell density normally have a punctate distribution of LIMD1 along cell junctions, reflecting the tension-dependent recruitment of LIMD1 to AJ under tension (Fig. A)(Ibar *et al.* , 2018). Induction of high level EGFP:AJUBA expression, using Dox, led to robust detection of AJUBA:GFP at AJ (Fig B). At the same time, detection of LIMD1 at AJ was significantly reduced (Fig A,B,S). This suggests that EGFP:AJUBA expressed at high levels has out-competed endogenous LIMD1 for association with α-catenin at AJ.

Induction of high level EGFP:WTIP expression, using Dox, led to detection of EGFP:WTIP both at AJ and in puncta dispersed throughout the cell (Fig H). Cytoplasmic puncta of WTIP were not observed when EGFP:WTIP was induced at low levels (Ibar *et al.* , 2018), but evidence that Ajuba family proteins can undergo phase separation has been reported previously (James et al., 2010; Wang et al., 2021), and the N-terminal halves of Ajuba family proteins are predicted to be intrinsically disordered (Rauskolb *et al.* , 2022; Wang *et al.* , 2021), which is a hallmark of proteins that form biomolecular condensates (Borcherds et al., 2021). Despite over-expressed EGFP:WTIP protein being distributed between AJ and cytoplasmic puncta, it also effectively removed endogenous LIMD1 from AJ (Fig. G,H,S).

***Over-expression of WTIP or AJUBA can lead to loss of LATS1 from AJ***

LIMD1 is required for recruitment of Lats kinases to AJ in MCF10A cells (Ibar *et al.* , 2018). However, all three Ajuba family proteins have been observed to associate with Lats kinases in co-immunoprecipitation experiments (Abe *et al.* , 2006; Das Thakur *et al.* , 2010; Jagannathan *et al.* , 2016; Reddy and Irvine, 2013; Sun and Irvine, 2013), suggesting that AJUBA or WTIP might be able to substitute for LIMD1. To investigate this possibility, MCF10A cells expressing high levels of EGFP:AJUBA or EGFP:WTIP were stained for expression of LATS1. In control MCF10A cells cultured at low density, LATS1 is predominantly localized to AJ (Fig. C,I,O)(Dutta *et al.* , 2018; Ibar *et al.* , 2018). High level expression of EGFP:AJUBA or EGFP:WTIP led to loss of LATS1 from AJ (Fig. D,J,T). In contrast, low level expression of these proteins does not displace Lats kinases (Ibar *et al.* , 2018). As a control, we also examined LATS1 localization when LIMD1 was over-expressed using 5 µg/mL Dox (prior experiments used 0.05 µg/mL Dox to match endogenous expression) (Ibar *et al.* , 2018). Over-expression of LIMD1 did not remove LATS1 from AJ (Fig. P,T). These observations indicate that despite localizing to AJ, EGFP:AJUBA or EGFP:WTIP are unable to recruit LATS1 there.

***Over-expression of WTIP or AJUBA increases YAP1 phosphorylation and decreases YAP1 nuclear localization***

Yap proteins are the key substrates of Lats kinases in the Hippo pathway. If the loss of LATS1 from AJ is functionally significant, then over-expression of AJUBA or WTIP could also impact phosphorylation of Yap proteins. This was investigated by using antisera detecting YAP1 and antisera detecting YAP1 phosphorylated at its major Lats phosphorylation site (Ser127, pYap) on western blots of lysates from cells over-expressing EGFP:AJUBA or EGFP:WTIP. Induction of either AJUBA or WTIP was sufficient to increase YAP1 phosphorylation (Fig. M,N). Phosphorylation of YAP1 results in a shift in localization from nuclear towards cytoplasmic, decreasing its activity as a transcriptional co-activator (Zheng and Pan, 2019). Consistent with this, over-expression of EGFP:AJUBA or EGFP:WTIP were also associated with a relative decrease in YAP1 nuclear localization (Fig. E,F,K,L,U). Conversely, over-expression of LIMD1 did not decrease YAP1 nuclear localization (Fig. Q,R,U).

**Conclusions**

The existence of three Ajuba family proteins in mammals raises the question of whether they act redundantly, or instead have distinct activities. Our investigations of over-expressed AJUBA and WTIP in MCF10A cells imply that these proteins are distinct from LIMD1 in lacking the ability to regulate Hippo signaling through tension at AJ. Not only are AJUBA and WTIP not normally required for regulation of Lats and Yap proteins in MCF10A cells (Ibar *et al.* , 2018), even when over-expressed they are unable to substitute for LIMD1 in Hippo pathway regulation.

Based on the structural similarity amongst Ajuba family proteins, and their shared ability to localize to AJ under tension, they would be expected to be able to compete with each other for binding to AJ. This competition would enable over-expressed AJUBA or WTIP to displace LIMD1, providing a simple explanation for the loss of LIMD1 from AJ. However, since AJUBA and WTIP can also associate with Lats kinases, they would have also been expected to substitute for LIMD1 at AJ, maintaining tension-dependent regulation of Lats kinases. Instead, we found that they fail to recruit LATS1 to AJ, resulting in increased Lats activity. While this is consistent with observations that knockdown of AJUBA or WTIP did not affect Lats or Yap activity (Ibar *et al.* , 2018), it is nonetheless surprising in light of observations that AJUBA or WTIP can associate with Lats kinases. However, evidence for association of AJUBA or WTIP with Lats kinases has been based on co-immunoprecipitation experiments using over-expressed proteins, only a fraction of which is at AJ. To explain the LIMD1-specific recruitment of Lats kinases to AJ, we thus propose that association with AJ alters the conformation of Ajuba family proteins in a way that enhances LIMD1-Lats association, but inhibits AJUBA and WTIP-Lats association, such that Lats association with AJ requires LIMD1.

AJUBA activity has been linked to a variety of cancers, and intriguingly has been found to act in some cases as a tumor suppressor, and in other cases as an oncogene (Jia et al., 2020; Schleicher and Schramek, 2021). Several mechanisms have been proposed for AJUBA’s links to oncogenesis, including effects on Hippo signaling. Notably, in some cases oncogenic activity of AJUBA has been correlated with increased YAP activity (Bi et al., 2018; Li et al., 2019; Zhang et al., 2018), whereas tumor suppressor activity of AJUBA has been correlated with decreased YAP activity (Gao et al., 2014; Liu et al., 2018; Tanaka et al., 2015). Additional studies will be required to decipher the complex links between AJUBA and Hippo signaling that impact oncogenesis, but our results suggest that competition with LIMD1 for Lats binding provides a potential mechanism by which AJUBA could increase Lats activity, and thereby suppress YAP-dependent oncogenesis.

## Methods

Cell Culture

MCF10A-EGFP-LIMD1 cells were cultured in DMEM/F-12 (Life Technologies) supplemented with 5% horse serum, 1% antibiotic-antimycotic, epidermal growth factor (20 μg/mL), insulin (10 μg/mL), hydrocortisone (0.5 μg/mL), cholera toxin (0.1 μg/mL) and puromycin (2 μg/mL) at 37⁰C and 5% CO₂ (Ibar et al., 2018). For immunostaining cells were grown on coverslips (Fisherbrand Microscope Cover Glass) coated with 0.6 mg/mL of collagen for 15 minutes at room temperature and washed with PBS. For the EGFP-LIMD1 overexpression experiment 60,000 cells per well were seeded in 24 wells plate, and 24 hours post-seeding cells were induced with 5 μg/mL of doxycycline for 24 hours.

Immunostainings and Imaging

Cells were fixed with 4% paraformaldehyde in phosphate-buffered saline (PBS) supplemented with 0.5 mM CaCl₂ and 1 mM MgCl₂ (PBS++) for 10 minutes at room temperature. Then cells were washed with PBS++ three times for 10 minutes each, followed by permeabilization with 0.5% Triton X-100 in PBS for 20 min. After blocking with 5% bovine serum albumin (BSA) in PBS for 1 hour, cells were incubated in primary antibodies diluted in 5% BSA in PBS solution overnight at 4⁰C. Primary antibodies used for immunostaining were: mouse anti-LIMD1 (1:500; EMD Millipore, 3F2/C6), rabbit anti-LIMD1 (1:500; Bethyl, A303-182A), rabbit anti-LATS1 (1:500; Cell Signaling, 3477S), rat anti-E-cadherin (1:500; Life Technology, 13-1900). Following the incubation with primary antibodies cells were washed three times with PBS for 5 minutes each, and incubated with secondary antibodies at room temperature for 2 hours. The secondary antibodies used for immunostaining were: Cy3 and Alexa Flour 647-conjugated (Jackson ImmunoResearch) in the dilution of 1:100. Upon incubation with secondary antibodies the cells were washed four times with PBS. Cell nuclei was stained with Hoechst 333342 (1 μg/mL; Invitrogen) and mounted with fluorescent mounting medium (Dako). Images were acquired on a Leica TCS SP8 confocal microscope system using a HC PL APO 63x/1.40 objective.

Western Blotting

Cells were lysed in 2x Laemmli (Bio-Rad) buffer supplemented with protease inhibitor (Roche) and phosphatase inhibitor cocktail (Calbiochem), sonicated for 5 seconds. Protein samples were denatured at 95⁰C for 5 minutes, centrifuged for 1 minute at max speed and loaded in 4% to 15% gradient gels (Bio-Rad). Primary antibodies used for the immunoblotting were: rabbit anti-phospho-YAP (S127) (1:1000; Cell Signaling, 4911S), rabbit inti-YAP (1:1000; Abcam, ab52771) and mouse anti-GFP (1:1000; Cell Signaling, 2955S). Mouse anti-α-tubulin (1:10000; Sigma, T6199) was used as a loading control. Blots were stained using fluorescent-conjugated secondary antibodies from LI-COR and visualized on an Odyssey Imaging System (LI-COR Biosciences).

Image Quantitation and Statistical Analysis

Confocal images were quantified using Volocity (Perkin Elmer) and Fiji (Schindelin et al., 2012) software. E-cad staining was used to define a junctional region for quantitation of LIMD1 and LATS1, while Hoechst staining was used to define a nuclear region for quantitation of YAP1 localization. Comparisons between control and experimental conditions were performed by unpaired t test on the log of the ratios, using Prism software (Graphpad).

## Reagents

EGFP:AJUBA, EGFP:WTIP and EGFP:LIMD1 expression vectors have been previously described (Ibar *et al.* , 2018) and deposited in Addgene.
